# Age Is Just a Number: Ontogenetic Conservation in Activation of Blood Clotting Factors VII, X, and XII by Caucasus Blunt-Nosed Viper (*Macrovipera lebetina obtusa*) Venoms

**DOI:** 10.3390/toxins16120520

**Published:** 2024-12-02

**Authors:** Katrina Kempson, Abhinandan Chowdhury, Aude Violette, Rudy Fourmy, Raul Soria, Bryan G. Fry

**Affiliations:** 1 Adaptive Biotoxicology Lab, School of the Environment, University of Queensland, St Lucia, QLD 4072, Australia; katrinakempson@outlook.com (K.K.); abhinandan.choudhury@uq.edu.au (A.C.); 2 Biomedical Science, Queensland University of Technology, Brisbane, QLD 4000, Australia; 3 Alphabiotoxine Laboratory Sprl, Barberie 15, 7911 Montroeul-au-bois, Belgium; aude.violette@alphabiotoxine.com (A.V.); info@alphabiotoxine.be (R.F.); 4 Inosan Biopharma, 28108 Madrid, Spain; rsoria@inosanbiopharma.com

**Keywords:** venom, antivenom, *Macrovipera*, ontogeny, evolution

## Abstract

This study examined the pathophysiological effects of venoms from neonate and adult specimens of the viperid snake *Macrovipera lebetina obtusa*, focusing on their ability to activate various blood clotting factors in human plasma. All venoms exhibited strong procoagulant properties. In concentration–response tests, the clotting potency of the neonate venoms fell within the range of their parents’ maximum clotting velocities and areas under the curve. Intriguingly, females were more potent than males within each age group, but this requires a larger sample size to confirm. Antivenom neutralization efficacy was equipotent across age groups. The venoms potently activated Factor X (FX) robustly, consistent with previous knowledge of this genus. For the first time, the ability to activate Factors VII (FVII) and XII (FXII) was identified in this genus, with FXII exhibiting particularly strong activation. The study found no significant ontogenetic variation in procoagulant venom potency on human plasma, convergent with the *Daboia* genus, the other large-bodied lineage within the Palearctic viperid clade. However, the activation of FXII and FVII reveals previously undocumented pathways in the procoagulant activity of these venoms, contributing to the broader understanding of venom evolution and its clinical impacts. These findings have implications for venom biodiscovery and the development of antivenoms, highlighting the complexity of clotting factor activation beyond traditional investigations that have myopically focused upon FX and prothrombin pathways, thereby underscoring the importance of exploring additional clotting factors.

## 1. Introduction

As a neglected tropical disease, snakebite is a critical human public health problem that has a conservative annual impact of 1.8–2.7 envenomations, >400,000 maims, and >100,000 deaths [1,2]. However, these numbers are well recognized as gross underestimations due to the poor/non-existent epidemiological records kept in some of the most affected regions [3,4,5]. In addition to life-threatening systemic effects, envenomations may produce severe local necrotic effects resulting in amputations [6,7]. Of particular concern is the highly dynamic nature of snake venom protein evolution, characterized by accelerated gene duplication and diversification [8,9,10,11,12,13,14,15,16,17], leading to differential pathophysiological actions with consequent impacts upon antivenom efficacy [18,19,20,21,22,23,24,25,26,27,28,29,30,31,32,33,34,35,36,37,38].

Extensive venom variation is a consequence of different toxins in a given species venom profile and can occur between all population dynamics including age (ontogenetic), sex, and locality groups [39,40,41,42,43,44,45,46,47,48,49,50,51]. Of these variables, ontogenetic variation can cause variations in antivenom response across the life-stages of a single snake. For example, the *Crotalus culminatus* neonate venom phenotype is procoagulant compared to the typical anticoagulant action of rattlesnake venoms, and consequently it is not neutralized by antivenom [48]. A particularly extreme case of ontogenetic variation concerns the Australian *Pseudonaja* species (brown snakes). The antivenom is made using the venom of the adults, which are diurnal specialists preying upon mammals with procoagulant venoms, while the neonates are nocturnal specialists preying upon lizards with neurotoxic venoms [41,52]. While differences in diet are the primary drivers of venom variation [11,53,54,55,56,57,58], venom variation may also result from differential expression in toxins used defensively. For example, the extensive variation in cytotoxicity in *Naja* species (cobras) and *Ophiophagus* species (king cobras) parallels the relative presence of aposematic markings and coloring [59]. Defensive toxin variation may even be ontogenetic, as seen in the cytotoxicity levels of *Ophiophagus* venoms. Juveniles, which have a concealed, semi-fossorial lifestyle, show lower levels of defensive cytotoxins compared to adults, who occupy exposed terrestrial niches and face higher predation risks [60]. The defensive pain-induction in cobra venoms has arisen convergently on multiple occasions, paralleling the evolution of spitting on three occasions [61].

Snake venoms may affect any part of the body reachable by the bloodstream or lymphatics system, causing pathophysiological alterations of blood biochemistry via either anticoagulant or procoagulant actions [62]. Hemorrhagic shock results from the anticoagulant blocking of the key components of the blood clotting cascade, leading to excessive bleeding, while, conversely, procoagulant venoms trigger the clotting cascade, resulting in stroke in prey animals but causing venom-induced consumptive coagulopathy (VICC) in the larger blood volumes of human victims [7,63,64]. Procoagulant venoms activate the zymogen form of blood-clotting enzymes, leading to the generation of endogenous thrombin, which, in turn, converts fibrinogen into fibrin clots [46,47,49,50,57,65,66,67,68,69,70,71,72,73,74,75,76,77,78,79,80,81].

This study investigated the relative variation in venom procoagulant potency between neonates and adults of the viperid species *Macrovipera lebetina obtusa*, members of a genus that has been documented as having venoms rich in blood clotting factor-activating metalloprotease enzymes [69,82,83,84,85,86,87]. This study also investigated the relative antivenom efficacy between neonate and adult venom phenotypes.

## 2. Results and Discussion

**Figure 1:**
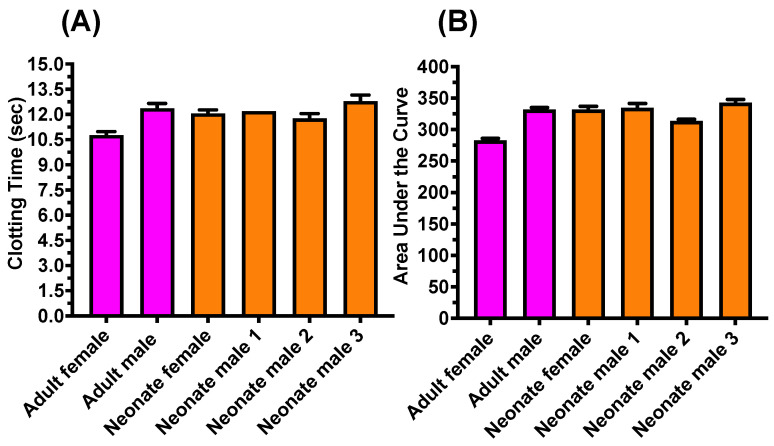
Plasma clotting: (**A**) velocity (time in seconds) at the highest venom concentration (20 μg/mL), whereby lower times equal greater potency, and (**B**) area under the curve (AUC) for an eight-point concentration–response curve (0.05, 0.125, 0.25, 0.66, 1.66, 4, 10, and 20 μg/mL), whereby smaller values equal greater potency. Data are (n = 3) calculated as mean ± standard deviation.

**Figure 2:**
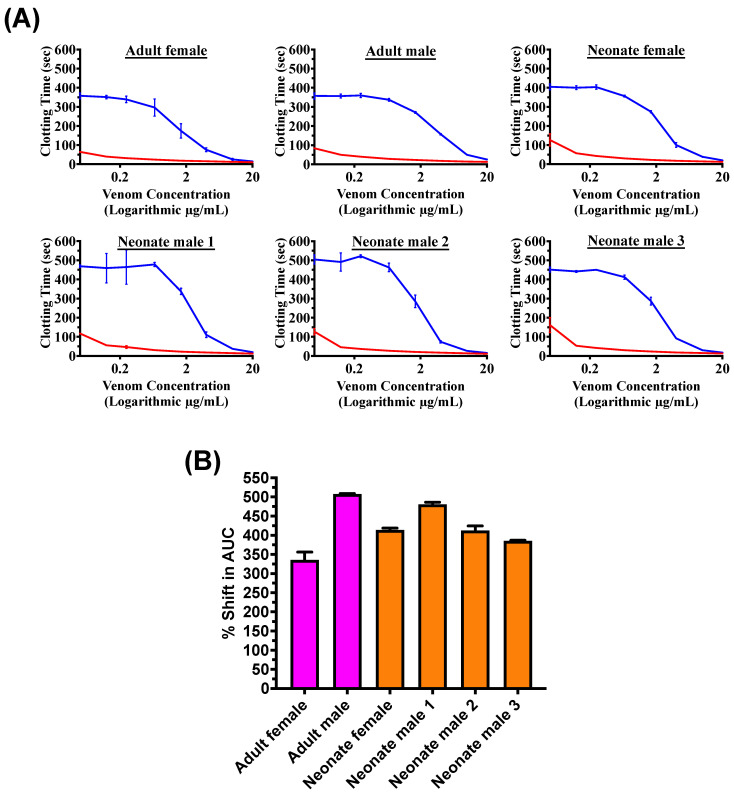
(**A**) An eight-point concentration–response curve (0.05, 0.125, 0.25, 0.66, 1.66, 4, 10, and 20 μg/mL in logarithmic view) for venom (**red**) and venom + Inoserp Europe ^TM^ antivenom (**blue**), where lower values indicate a greater venom effect, and (**B**) a relative shift in the area under the curve (AUC), where higher values indicate greater antivenom efficacy; the data shows there was no difference in the antivenom neutralizing effects of the venoms of adults and neonates of either sex. Data are (n = 3) calculated as mean ± standard deviation.

Tests for effects upon clotting revealed all venoms to be potently procoagulant, with all neonates falling within the range of their parents both in terms of maximum velocity (Figure 1A) and the area under the curve obtained from an eight-point concentration–response curve (Figure 1B). All venoms were well neutralized by the Inoserp^TM^ Europe antivenom, consistent with previous work showing the strong effect that this antivenom has upon *Macrovipera* venoms [69] (Figure 2A). There was no difference between adults and neonates, with all neonates falling within the range of adults (Figure 2B).

**Figure 3:**
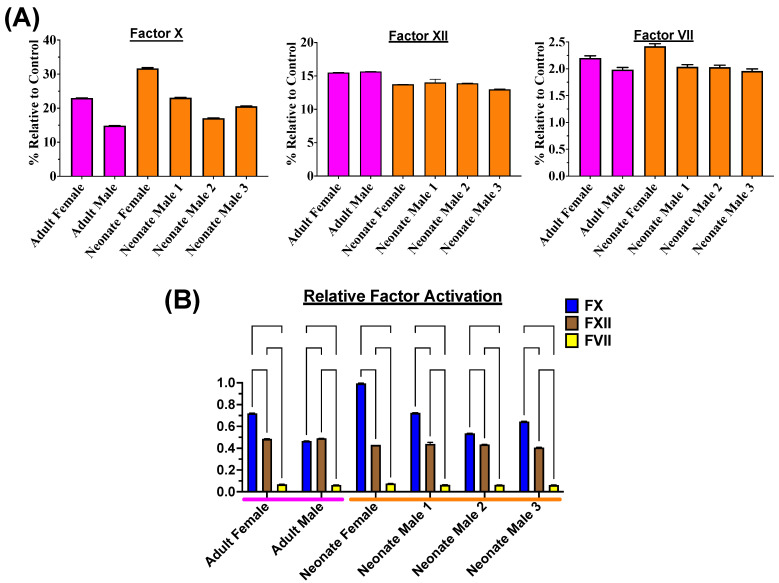
(**A**) The activation of blood clotting Factors X, XII, and VII, as measured by fluorescence relative to control enzymes (Xa, XIIa, and VIIa, respectively). (**B**) The relative activation of Factor X, FXII, and FVII normalized against the most potent activation (neonate female on Factor X). Statistics are Brown–Forsythe and Welch ANOVA tests with post hoc Dunnett’s T3 multiple comparisons. Data are (n = 3) calculated as mean ± standard deviation.

Venoms of all age groups and both sexes were shown to be potent activators of FX with negligible effects on prothrombin (Figure 3), which is consistent with previous studies [69]. The venoms also had no activation effects upon Factors IX or XI. However, for the first time for this genus, the abilities to activate Factors VII and XII were revealed. While FVII was not strongly activated, FXII was potently activated (Figure 3). Intriguingly, for Factor X activation, both the adult and neonate females were more potent than the males of their respective age group, and both neonate sexes were more potent than their corresponding adult. However, as the effect was not dramatic, future work with a larger sample size is necessary to ascertain if this is a consistent trend. Regardless, as FX is but one of three factors activated, these low-level differences in FX activation did not affect the overall impact upon the net procoagulant effect seen in Figure 1.

The strong procoagulant actions of the venoms in this study were congruent with previous studies that showed a strong procoagulant action is characteristic of the venom for the genus *Macrovipera* [69], as well as that their venom contains snake venom metalloproteases (SVMPs) demonstrated as having the ability to activate Factors X and V [88,89]. Congruent with these actions, the clinical effects following *Macrovipera* envenomation to humans include severe local tissue damage and lethal systemic coagulopathy [90,91,92]. Aligning with this, proteomic studies of *Macrovipera* venoms have shown them to be rich in SVMPs [82,83,84,86,87,93].

Predatory ecology is the major variable that influences the relative shift between neonate and adult venoms. If there is a major difference, this suggests a corresponding difference in prey type, prey escape potential, or both [48,94]. Reciprocally, if there is no significant difference across age groups, this indicates predatory ecology conservation [95,96]. Within the Palearctic clade consisting of the genera *Daboia*, *Macrovipera*, *Montivipera*, and *Vipera*, venom variations between neonates and adults has only been previously investigated in only two of the genera (*Daboia* and *Vipera*) and a handful of species. While *Daboia russelii* venoms have been shown to ontogenetically vary in some constituents [97], the effects upon human plasma are conserved, indicative of conservation of procoagulant SVMPs [79]. Within *Vipera*, proteomics studies revealed juvenile *V. latastei* to have higher concentrations of SVMP enzymes [98], the toxin type responsible for procoagulant toxicity in this genus [99]. Consistent with this being a generalized ontogenetic trend within this genus, juvenile *V. ammodytes meridionalis* animals were shown to have higher SVMP-driven procoagulant potency and be stronger activators of FX [100].

*Daboia* and *Macrovipera* represent independent evolutions of gigantism within the Palearctic clade, being much larger than the diminutive *Montivipera* and *Vipera* species, which retain the small-bodied ancestral condition (with even exceptional specimens of the largest derived species *Montivipera xanthina* and *Vipera ammodytes* being smaller than the average *Daboia* or *Macrovipera*) [101]. Paralleling the morphological diversification, the larger-bodied species are more potently procoagulant [99]. With the contribution of this study’s data to the body of knowledge, an interesting trend is emerging stating that not only are the venoms of *Daboia* and *Macrovipera* more procoagulantly potent, but also they do not display strong ontogenetic variation in potency. This is in contrast to the genus *Vipera*, where proteomics studies showed juvenile venoms to have higher concentrations of SVMP enzymes [98]. This is the toxin type responsible for procoagulant toxicity in this genus [99]. Bioactivity testing confirmed a strong ontogenetic signal for procoagulant potency in *V. ammodytes meridionalis* venoms [100]. Therefore, this study has generated a testable hypothesis for future work investigating ontogenetic venom variation as a factor of size in additional *Daboia*, *Macrovipera*, *Montivipera*, and *Vipera* species. This in turn will contribute to the broader evolutionary theory regarding venom variation as a function of changes in predatory ecology, as has been noted in other species. For example, within a clade of Australian elapid snakes, there is a dramatic difference in relative ontogenetic patterns between the large-sized *Oxyuranus* species (taipans) and those of their smaller sister genus *Pseudonaja* (brown snakes). *Oxyuranus* venoms do not ontogenetically vary, consistent with these snakes being mammal specialists at all ages [41,102,103]. In contrast, *Pseudonaja* venom have marked changes in venom biochemistry, with the lizard specialist neonates being neurotoxic, while the mammal specialist adults are procoagulant [41,52].

This study has implications beyond ontogenetic venom variations and has attained a significant biodiscovery element through the documentation of FXII as potently activated, along with FVII to a much lesser but still notable degree. This contributes to the growing body of evidence suggesting that clotting factors beyond just FX and prothrombin may be important for venom-induced procoagulant pathophysiological actions. Historically, only FX and prothrombin activation have been tested for, leading to an abundance of papers on these effects [65,69,70,71,73,74,75,100,104,105,106,107,108,109,110,111,112,113,114,115,116,117,118]. In contrast, investigations on the ability of venom to activate clotting factors have been scarce.

Studies that have investigated factor activation beyond FX and prothrombin include the Asian natricine snakes in the genus *Rhabdophis*; Australian elapid snakes in the *Hoplocephalus*, *Notechis*, *Oxyuranus*, *Pseudonaja*, and *Tropidechis* genera; and the Central American pit viper species *Porthidium volcanicum* in the genus *Rhabdophis* have long only considered these species’ ability to activate Factor X and prothrombin [107,112,118], but when examined for other factor activations, thy have been shown to activate FVII >> FIX > FXII > prothrombin > FX [68]. As such, the only two factors historically studied for *Rhabdophis* venoms (FX and prothrombin) were in fact shown to be the least activated [68]. While prothrombin activation has long been stated as the mechanism by which Australian elapid snake venoms are procoagulant, this action had in fact only been tested for venoms from the genera *Hoplocephalus*, *Notechis*, *Oxyuranus*, *Pseudonaja*, and *Tropidechis* [119,120,121,122,123,124,125,126]. However, when prothrombin activation was compared to Factor VII activation across the full taxonomical range of Australian elapid snake venoms, it was shown that FVII activation was the basal trait, while only some derived lineages were capable of activating prothrombin [127]. The viperid snake *Porthidium volcanicum* was shown to be unique in its genus as the only procoagulant species, which was accomplished through the activation of FVII > FXII > FXI > FX [72]. Clotting factor activation extended to the venoms of lizards in the *Heloderma* genus [128]. FVII and FXII were shown to be the two major pathophysiological targets. There was also a strong ontogenetic signal. *H. suspectum* venom displayed a phenotypic venom profile, being highly similar to the neonate *H. exasperatum* venom in strongly activating FXII [128]. In contrast, FVII was most strongly activated by adult *H. horridum* venom [128]. These biochemical variations underscore not only the evolutionary novelty of reptile venoms but also the diverse clinical effects that they produce and their endless biodiscovery potential.

## 3. Materials and Methods

### 3.1. Venom Stock Preparation

Venom stock preparation was carried out as previously described by us [100,128]. Venom work was conducted with University of Queensland Biosafety Committee Approval # IBC/134B/SBS/2015 and Animal Ethics Approval 2021/AE000075. Six lyophilized *Macrovipera lebetina obtusa* venoms were provided by the Alphabiotoxine Laboratory (Belgium). Samples included venom from two adult individuals (male and female, caught in Georgia, USA) and their offspring (four neonates, milked at 3 months of age). Lyophilized venom samples were stored in a −80 °C freezer until use. Venom liquid stocks for running assays were prepared to preserve enzymatic activity, reconstituting 1 mg into 1 mL of 50% double deionized water/50% glycerol. A Thermo Fisher Scientific NanoDrop 2000 UV–Vis Spectrophotometer (Thermofisher, Sydney, NSW, Australia) was used to determine protein concentrations. Moreover, 50% double deionized water/50% glycerol liquid venom stocks were stored in a −20 °C freezer.

### 3.2. Plasma Coagulation Assays

Plasma coagulation assays were conducted as previously described by us [100,128]. Human plasma work was performed with University of Queensland Human Ethics Approval #2016000256 and Biosafety Approval #IBC134BSBS2015. Human platelet-poor plasma (3.2% citrated) was supplied under research approval #16- 04QLD-10 granted by the Australian Red Cross (44 Musk Street, Kelvin Grove, QLD 4059, Australia). Plasma was aliquoted into 1.5 mL volumes, flash-frozen in liquid nitrogen, and stored at −80 °C until required. For testing, plasma was defrosted in a 37 °C water bath for 5 min before use.

### *3.3.* Coagulation Curves

Coagulation curves were conducted as previously described by us [100,128]. Plasma samples were thawed in a 37 °C water bath for 5 min prior to testing. A Stago STA-R Max hemostasis analyzer (Stago, Asnières sur Seine, France) was used to test the ability of venoms to clot human plasma at eight concentrations ((0 μg/mL, 10 μg/mL, 4 μg/mL, 1.6 μg/mL, 0.66 μg/mL, 0.25 μg/mL, 0.125 μg/mL, and 0.05 μg/mL), with all concentrations run in triplicate. As previously described by us [100,128], 1 mg/mL of venom stock was diluted with OK buffer to 0.1 mg/mL and placed into the analyzer. For the 20 μg/mL concentration, 50 μL of 0.025 M CaCl2, 50 μL of phospholipid solubilized in 25 μL OK buffer, and 50 μL of 0.1 mg/mL venom were automatically pipetted into a cuvette and incubated for 120 s at 37 °C. Following incubation, 75 μL of human plasma was added to the cuvette, and clotting time was measured using a mechanical, viscosity-based system. For additional concentrations, the volumes of venom and OK buffer added to the cuvette were automatically adjusted by the Stago STA-R Max hemostasis analyzer. The final cuvette volume for all reactions was 250 μL.

### 3.4. Antivenom Neutralization Studies

Antivenom neutralization studies were conducted as previously described by us [100,128]. To test the efficacy of antivenom in neutralizing the coagulotoxic activity of *Macrovipera lebetinus obtusa* venom, antivenom assays were performed on a Stago STA-R Max hemostasis analyzer. The antivenom tested was Inoserp^TM^ Europe (Inosan, Biopharma, S.A., Madrid, Spain; Viteria Labs, S.A. de C.V., Mexico) (lot # 9IT03006), a 22.5 mg/mL F(ab′)2 antivenom made using an immunizing mixture consisting of *Macrovipera lebetina cernovi*, *M. l. obtusa*, *M. l. turanica*, *M. schweizeri*, *Montivipera xanthina*, *Vipera ammodytes*, *V. aspis*, *V. berus*, and *V. latastei*. As previously described by us [100], antivenom was diluted with OK buffer to a concentration of 5%. The same procedure as used in the plasma coagulation assays (Section 3.3) was followed, except that 25 μL of OK buffer was replaced with 25 μL of 5% antivenom, leading to a final antivenom cuvette reaction concentration of 0.5%.

### 3.5. Clotting Factor Activation Assays

Clotting factor activation assays were conducted as previously described by us [100,128]. As previously described by us [100], to detect the activation of clotting factors (Factor VII, IX, X, XI, XII, and prothrombin) and compare the relative ability of factor activation between neonate and adult *Macrovipera lebetinus obtusa* venom, clotting factor activation assays were performed with Fluoroskan Ascent (Thermo Scientific, Vantaa, Finland). A Hamilton Vantage Liquid Handling System (USA) automatically plated reagents into 384-well plates (black, lot#1171125; Nunc Thermo Scientific, Rochester, NY, USA). Plates were then manually loaded into the Fluoroskan Ascent, and measurements were started. The Fluoroskan Ascent automatically pipetted 70 μL of buffer, which contained 5 mM of CaCl_2_, 150 mM of NaCl, 50 mM of Tris-HCl (pH 7.3), and Fluorogenic Peptide Substrate (ES011Boc-Val-Pro-Arg-AMC. Boc: t-Butyloxycarbonyl; 7-Amino-4-methylcoumarin; R & D systems, Cat# ES011, Minneapolis, MN, USA) in a 500:1 ratio, to each well to start the reaction. The plate was warmed up at 37 °C and shaken for 3 s in Fluoroskan Ascent before each measurement. Fluorescence measurements were taken once a second for 300 s at 390 (excitation)/460 nm (emission) by Ascent Software v2.6 (Thermo Scientific, Vantaa, Finland). To obtain final results, the subtraction of “venom without zymogen” values from “venom with zymogen” values was performed, which nullified artificial increases in the fluorescence values caused by venoms that work directly on the substrate. Finally, the results from the subtractions were normalized as a percentage relative to the positive control (activated factors/enzyme (note: FXII was activated by using Kaolin and that solution was used as control)) by processing them in Excel and then analyzing them in GraphPad PRISM 8.1.1 (GraphPad Prism Inc., La Jolla, CA, USA).

### 3.6. Statistical Analyses

Statistical analyses were conducted as previously described by us [100,128]. GraphPad PRISM 8.1.1 (GraphPad Prism Inc., La Jolla, CA, USA) was used to perform statistical analyses. For the plasma clotting time of venom and venom incubated with antivenom, an area under the curve (AUC) was generated based on venom concentration–clotting time curves. To test and compare antivenom efficacy, an X-fold shift was calculated with the following formula:

$$\%\;shift=\left(\frac{AUC\;of\;\left(venom+antivenom\right)}{AUC\;of\;venom}-1\right)\;*\;100$$

The value of the % shift indicates the neutralization of venom activity achieved by antivenom. A percentage shift of 0 indicates no neutralization, while a value above 0 indicates neutralization. The statistically significant results in percentage AUC shift were classed as *p* < 0.05.

## Data Availability

All data are presented in the figures.
